# The role of male partner in utilization of maternal health care services in Ethiopia: a community-based couple study

**DOI:** 10.1186/s12884-019-2176-z

**Published:** 2019-01-14

**Authors:** Bedru Hussen Mohammed, Janice Mary Johnston, Dana Vackova, Semira Mehammed Hassen, Huso Yi

**Affiliations:** 1 School of Public Health, The University of Hong Kong, Hong Kong, Hong Kong SAR; 2 School of Nursing, The University of Hong Kong, Hong Kong, Hong Kong SAR; 3 The Jockey Club School of Public Health and Primary Care, The Chinese University of Hong Kong, Hong Kong, Hong Kong SAR

**Keywords:** Male partner involvement, Maternal health care services, Prevention of mother-to-child transmission of HIV, Antenatal care, Addis Ababa, Ethiopia

## Abstract

**Background:**

Ethiopia has recorded substantial progress in maternal health recently. However, poor utilization of maternal health care services is challenging further improvement. Although male partners are decision-makers in households, the impact of their involvement on maternal health care services has not been well studied. Thus, the objective of this study was to examine the association between male partners’ involvement in maternal health care on utilization of maternal health care services.

**Methods:**

A community-based cross-sectional study was conducted on male/female couples with a baby less than 6 months old (*N* = 210) in Addis Ababa, Ethiopia. The main independent variable of the study was male partners’ involvement in maternal health care. Two structured questionnaires were used to collect the data from men and women. Bivariate and multivariate logistic regression models were used to examine the relationship between the dependent and independent variables.

**Results:**

Mean age in years was 28.7 (SD = 5.4) for women and 36.2 (SD = 8.8) for men. Half of the men (51.4%) have accompanied their partner to antenatal care (ANC) at least once. However, only 23.1% of them have physically entered the ANC room together. Overall involvement of male partners was poor in 34.8% of the couples (involved in two or fewer activities). After controlling for other covariates, the odds of having 1st ANC visit within the first trimester of pregnancy and skilled delivery attendant at birth were higher in women whose male partners took time to know what happened during ANC visits (AOR = 1.93; 95%CI = 1.04–3.60; AOR = 2.93; 95%CI = 1.24–5.6.90, respectively). Similarly, the odds of having at least one ANC visit, first ANC visit within twelve weeks, HIV testing, skilled birth attendant, and birth in a health facility were higher in couples with higher overall male partner involvement.

**Conclusion:**

The study demonstrated significant associations between male partners’ involvement in maternal health care and utilization of some maternal health care services by female partners.

## Background

The Millennium Development Goals (MDGs) report (2015) shows that there was a substantial improvement in maternal and child health globally as measured by under-five mortality rate (declined by 53%) and maternal mortality ratio (declined by 44%) between 1990 and 2015 [1]. Despite this progress, maternal mortality ratio remains unacceptably high in low-income countries particularly in sub-Saharan Africa [2]. In Ethiopia, maternal mortality ratio remains high, 412 per 100,000 live births [3].

Ethiopia has now joined the current global effort as embodied in the new Sustainable Development Goals (SDGs) which seek to keep the spotlight on the unfinished agenda of ending preventable maternal, newborn and child mortality [1]. Poor utilization of maternal health care services highlight the challenges to further improving maternal and child health [4].

The 1994 International Conference on Population and Development in Cairo and the 1995 International Conference on Women in Beijing called global attention to the importance of involving men in maternal and child health [5, 6]. Men are an important stakeholder and should be considered as half of the equation in maternal and child health [7, 8]. Even though men have important decision-making role related to maternal and child health issues, in many sub-Saharan Africa countries including Ethiopia, maternal and child health is viewed as a woman’s affair [9, 10].

Several studies have reported the positive impact of male partner’s involvement on maternal health care services [11–31]. Male partner’s involvement in maternal and child health care service is reported to be associated with increased uptake of maternal health care such as antenatal care (ANC), the probability of having facility-based delivery, contraception use, decreased mother-to-child transmission of HIV (MTCT), and decreased post-abortion recovery time [11, 32, 33]. Some studies also showed that when couples receive counseling together, there is a better use of infant feeding methods [34, 35], have a higher acceptance of HIV testing [36] and they are more likely to adhere to Antiretroviral therapy (ART) [34, 35]. Aluisio et al. reported that male partner’s involvement in maternal health care is associated with low risk of HIV infection in infants of HIV infected women and greater HIV free survival [37].

Other studies reported that male partner’s involvement in maternal health care services could occasionally lead to domestic discord, emotional and physical intimate violence against women, disruptions of family relationships, loss of economic support to women, and blame and abandonment of women [11, 37–42]. Njunga and Blystad [40] described a situation in Malawi whereby the PMTCT program was known as the ‘divorce program’ as the request for partner disclosure by the program led to numerous family dissolutions.

Despite the potential benefits, male partner’s involvement in maternal and child health in Ethiopia is low and regionally variable. Only 28.1% of male partners in Addis Ababa participated in four or more of six PMTCT activities [43]; 40.1% in Gondar were involved in HIV counselling and testing during their wife’s pregnancy [44]; 20% in Mekelle accompanied their pregnant wives to the maternal health care services [45]; and 53% in Arba Minch reported involvement in PMTCT programs [46].

The evidence above illustrates the potential benefit of male partner’s involvement in maternal and child health programs. Attempts to examine male partner’s involvement in maternal and child health services in Ethiopia have been very limited, relying largely on socio-demographic and maternal characteristics from surveys on women and, less so, from men who attended ANC services with their partners [43–47]. The current study intended to expand this individual level analysis and explore the impact of male partner’s involvement on the female partner’s use of maternal health care services from the couple’s perspective. The study hypothesized that there was no association between male partner involvement and their partners’ utilization of maternal health care services. It sought to determine whether, after controlling for individual level covariates, male partner’s involvement is beneficial to maternal health care services utilization among couples in Addis Ababa, Ethiopia.

## Methods

### Study design and sampling

The study sample was taken from a larger community-based cross-sectional study examining utilization of maternal health care services in Addis Ababa-Ethiopia, 2014. Two hundred ten couples who recently had a baby and have lived in Addis Ababa for at least one year. The study methods and data collection technique have been described previously [42].

**Study measures:** the ***primary outcome variable*** was female partner’s maternal health care services utilization. The WHO recommendation suggested that pregnant women should have their first ANC in the first 12 weeks’ gestation, with subsequent seven more contacts taking place at 20, 26, 30, 34, 36, 38 and 40 weeks’ gestation [48]. Thus, the study used six items to measure the outcome variable. The items are, having [1]: at least one ANC attendance during the last pregnancy [2], the first ANC appointment in the first twelve weeks of pregnancy [3], four (or more) ANC visits throughout their pregnancy [4], tested for HIV during the pregnancy [5], skilled birth attendant, and [6] delivered in a health care facility (clinic or hospital).

Each item was equally weighted in the construction of maternal health care utilization scale. The score was the sum of each item. Cronbach’s alpha of the scale was 0.706, which shows acceptable internal consistency.

The ***main explanatory variable*** was male partner involvement in maternal health care services. A scale derived from items identified in other studies and where each item was equally weighted (scores ranged from 0 to 8), was adopted to measure male partner’s involvement in maternal health care services [49, 50]. Male partner was considered fully involved if he has [1]: initiated a discussion about PMTCT with his partner [2]; requested his partner to be tested for HIV during pregnancy [3]; took time to find out what went on during partner’s ANC visits [4]; reminded his partner’s ANC follow-up schedule [5]; covered medical/transport costs of partner’s ANC follow-up [6]; accompanied partner to ANC clinic at least once [7]; physically entered the ANC room together with his partner; and [8] was counseled and tested for HIV during partner’s pregnancy.

Female partners’ confirmation of male partners’ involvement as per the eight-item scale described above was used to corroborate male partner response. Cronbach’s alpha of the scale was 0.748 indicating an acceptable internal consistency. Based on their involvement scale score male partners were divided into three groups; low male partner involvement (involved in less than three activities), moderate male partner involvement (involved in three to six activities), and high male partner involvement (involved in more than six activities).

Other covariates that are included in the analyses are; age, parity, educational status, employment, and household income.

### Statistical analyses

The study used frequencies and proportions to characterize the study participants. Chi-square test was used to test statistical significance of maternal health care services use differences, based on the explanatory variables. Explanatory variables found to be statistically significant in bivariate logistic regression analysis were entered into multivariate logistic regression analysis for adjustment of confounding between independent and dependent variables.

One model was estimated for each component of maternal health care and one for overall maternal health service use. The study assessed the association between male partner’s involvement and maternal health care services utilization, while controlling for the other covariates. Adjusted odds ratios with 95% confidence intervals were used to estimate the power of the association. The Statistical Package for Social Sciences (SPSS version 24.0 for Windows; SPSS Inc., Chicago, IL, USA) used for data analysis.

## Results

### Socio-demographic and relationship characteristics

Two hundred ten couples participated in the study. Female partners were significantly younger (Mean age_female_ = 28,7 (SD = 5.4) vs Mean age_male_ = 36.2 (SD = 8.8), with lower levels of education (15% for no formal education) and lower levels of employment (48% unemployed) than male partners (Table 1). The proportion of women who had no formal education was 15.2% (*n* = 32), which is higher than their male counterparts (9.5%; *n* = 20). About 32% (*n* = 67) of women were employed, while only 4.7% (*n* = 10) of the male partners were unemployed or retired. Most of the couples (96.7%; *n* = 203) were married with mean relationship duration of 6.9 years and only 3.3% (*n* = 7) were cohabiting. The relationship type that is predominant in the study couples was monogamy, accounted for 92.4% (*n* = 194). The mean (SD) number of children in the relationship was 2.2 (1.26). In 12% (*n* = 5.7) of couples, women are not involved in household decisions including their own personal health care service use.

**Table 1:** Socio-demographic and relationship characteristics of participants, Addis Ababa, 2014 (*N* = 210)

Background Characteristics	Male Partner n (%)	Female Partner n (%)	Couple n (%)
Age in years			
18–25	7 (3.3)	50 (23.8)	
26–35	113 (53.8)	131 (62.4)	
≥ 36	90 (42.9)	29 (13.8)	
Age gap of couples			
Male younger or same age as women			21 (10.0)
Male older by 1–5 years			73 (34.8)
Male older by 6–10 years			64 (30.4)
Male older by more than 10 years			52 (24.8)
Ethnicity			
Amhara	77 (36.6)	78 (37.1)	
Oromo	48 (22.9)	46 (21.9)	
Tigre	26 (12.4)	27 (12.9)	
Other	59 (28.1)	59 (28.1)	
Religion			
Orthodox Christian	129 (61.4)	128 (61.0)	
Muslim	55 (26.2)	56 (26.6)	
Other	26 (12.4)	26 (12.4)	
Education			
No formal education	20 (9.5)	32 (15.2)	
Primary school	50 (23.8)	80 (38.1)	
Secondary and above	140 (66.7)	98 (46.7)	
Employment			
Employed	200 (95.3)	67 (31.9)	
Unemployed	10 (4.7)	143 (68.1)	
Monthly income (ETB^a^)			
Low income (</=1000ETB)			73 (34.8)
Middle income (1001-2000ETB)			70 (33.3)
High income (>2000ETB)			67 (31.9)
Couples relationship duration			
Less or equal to 4 years			91 (43.3)
More than 4 years			119 (56.7)
Relationship type			
Polygamy			16 (7.6)
Monogamy			194 (92.4)
Relationship status			
Married with legal certificate			98 (46.7)
Married without legal certificate			105 (50.0)
Cohabiting (not married)			7 (3.3)
Weekly mass media exposure			
No exposure to all	15 (7.1)	31 (14.8)	
Exposed to one type	40 (19.0)	77 (36.7)	
Exposed to two types	77 (36.7)	78 (37.1)	
Exposed to all three types	78 (37.1)	24 (11.4)	
Couples’ access to any one of the mass medias at least once a week			
Both partners exposed			188 (89.5)
Only one partner exposed			16 (7.6)
Neither partner exposed			6 (2.9)
Parity			
one		66 (31.4)	
2–3		116 (55.2)	
4 or more		28 (13.3)	
Women’s decision making autonomy			
No involvement in all		12 (5.7)	
Involved in one		26 (12.4)	
Involved in two		31 (14.8)	
Involved in three		43 (20.5)	
Involved in all four		98 (46.7)	
IPV against women			
No			52 (24.8)
Yes			158 (75.2)

^a^*ETB* Ethiopian Birr (1 USD ~ 19 ETB at the time), *IPV* intimate partner violence (emotional, sexual, physical or controlling) against women occurred during the current relationship; Weekly mass media exposure = access to TV, Newspaper or Radio at least once a week

Women’s decision making autonomy = women’s participation alone or jointly in decisions regarding their personal health care, large and daily household purchases and family or relatives visit

Table 1 Socio-demographic and relationship characteristics of participants, Addis Ababa, 2014 (*N* = 210).

### Utilization of maternal health care services

For their most recent childbirth, 4.8% (*n* = 10) of the women had no ANC at all, 35.2% (*n* = 74) had four or more ANC visits, and only 49.0% (*n* = 103) had their first ANC visit within the first trimester of their pregnancy. Mean gestational age at first ANC visit was 3.5 months (SD = 1.29). However, 77.6% (*n* = 163) of the women gave birth in a health facility and 85.7% (*n* = 140) of them had a skilled healthcare provider.

### Male partner involvement

**Fig. 1:**
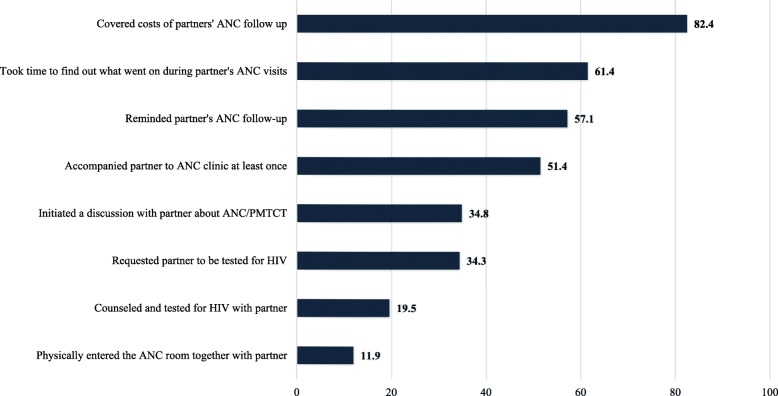
Male partner involvement in maternal health care services among couples; Addis Ababa, 2014 (*N* = 210)

Although most (82.4%; *n* = 173) male partners paid ANC related cost of their partners and 51.4% (*n* = 108) accompanied their partner to ANC at least once, only 11.9% (*n* = 25) of male partners entered to the ANC room with their partners for consultation (Fig. 1). The proportions of male partners who had initiated a discussion about ANC and/or PMTCT, and counseled and tested for HIV with their partners were 34.8% (*n* = 73) and 19.5% (*n* = 41), respectively. Overall involvement of male partners showed that only 9% (*n* = 19) have been involved in seven or all eight of the activities, while 56.2% (*n* = 118) were moderately (in three to six activities) involved and 34.8% (n = 73) reported low involvement (in two or fewer activities).

### Association between male partner involvement and women’s utilization of maternal health care services

Results from the binary logistic regression models for the association between each of the male partners’ involvement activities and the maternal health care services utilization indicators, while controlling for covariates are presented in Table 2.

**Table 2:** Logistic regression AOR for the association between male partners’ involvement and women’s utilization of maternal healthcare services, Addis Ababa, Ethiopia, 2014 (N = 210)

MPI	At least one ANC	1st ANC within 1st trimester	Four or more ANC	Tested for HIV	Skilled delivery attendant	Delivery in health facility	Utilized all services ^a^
	AOR^b^ (95% CI)	AOR (95% CI)	AOR (95% CI)	AOR (95% CI)	AOR (95% CI)	AOR (95% CI)	AOR (95% CI)
Initiated a discussion with partner about ANC/PMTCT	1.47 (0.28–7.81)	1.21 (0.66–2.22)	0.83 (0.42–1.63)	2.85 (0.99–8.23)	2.49 (0.87–7.11)	2.28 (0.99–5.28)	0.77 (0.37–1.62)
Requested partner to be tested for HIV	3.10 (0.35–27.15)	1.82 (0.97–3.38)	0.70 (0.35–1.40)	2.18 (0.75–6.35)	1.66 (0.60–4.55)	1.56 (0.68–3.56)	0.82 (0.39–1.71)
Took time to find out what went on during partner’s ANC visits	5.17 (1.19–22.48)*	1.93 (1.04–3.60)*	0.82 (0.41–1.62)	2.15 (0.91–5.07)	2.93 (1.24–6.9)*	1.95 (0.93–4.08)	0.77 (0.37–1.59)
Reminded partner’s ANC follow-up	3.28 (0.77–13.90)	1.68 (0.93–3.05)	0.84 (0.43–1.64)	2.36 (1.01–5.51)*	3.06 (1.30–7.24)*	1.30 (0.64–2.65)	0.79 (0.38–1.64)
Covered costs of partners’ ANC visits	2.72 (0.57–12.95)	2.69 (0.99–6.64)	1.25 (0.48–3.25)	1.27 (0.81–15.34)	2.34 (0.83–6.57)	1.35 (0.50–3.63)	1.26 (0.44–3.59)
Accompanied partner to ANC clinic at least once	5.49 (1.07–28.20)*	1.78 (0.98–3.22)	1.63 (0.83–3.18)	2.95 (1.25–7.00)*	1.85 (0.80–4.26)	1.15 (0.57–2.34)	1.68 (0.80–3.52)
Physically entered the ANC room with partner	0.94 (0.10–8.98)	1.01 (0.49–2.44)	1.29 (0.49–3.36)	1.81 (0.35–9.23)	1.20 (0.81–1.77)	7.58 (0.92–62.20)	1.02 (0.36–2.86)
Counseled and tested for HIV with partner	2.03 (0.21–19.37)	1.04 (0.48–2.22)	0.74 (0.31–1.76)	2.32 (0.58–9.33)	1.41 (0.42–4.70)	1.62 (0.56–4.65)	0.69 (0.27–1.72)
Overall MPI scale score	1.61 (1.05–2.45)*	1.19 (1.03–1.39)*	0.98 (0.83–1.15)	1.52 (1.18–1.96)**	1.44 (1.13–1.84)*	1.22 (1.01–1.48)*	0.97 (0.82–1.15)

*MPI* Male partners’ involvement, *ANC* antenatal care; ^a^ women utilized all maternal healthcare services (had four or more ANC visits starting 1st ANC within 1st trimester, tested for HIV and delivered in a health facility with a skilled delivery assistant); ^b^ models adjusted for women’s age, parity, intimate partner violence against women, women’s educational status, women’s employment status, and household monthly income; ∗ *p* < .05; ∗∗ = *p* < .01

Women whose partners accompanied them for their ANC visit (AOR = 5.49; 95%CI = 1.07–28.20) and whose partner took time to ask what went on during partner’s ANC visits (AOR = 5.17; 95%CI = 1.19–22.48) were more likely to have at least one ANC visit during their pregnancy. Similarly, women whose partners took time to know what happened in their ANC visits were more likely to have their 1st ANC visit within the first trimester (AOR = 1.93; 95%CI = 1.04–3.60).

The likelihood of having HIV test during pregnancy was higher in women whose male partners remind of their ANC visits (AOR = 2.36; 95%CI = 1.01–5.51) and accompanied for ANC visits (AOR = 2.95; 95%CI = 1.25–7.00). The odds of having skilled birth attendant was higher in women with male partners who took time to know what happened during ANC follow-up (AOR = 2.93; 95%CI = 1.24–5.6.90) and reminded their ANC follow-up (AOR = 3.06; 95%CI = 1.30–5.7.24).

Lastly, the odds of having at least one ANC (AOR = 1.61; 95%CI = 1.05–2.45), first ANC visit within first trimester (AOR = 1.19; 95%CI = 1.03–1.39), having HIV testing (AOR = 1.52; 95%CI = 1.18–1.96), delivery with the help of skilled birth attendant (AOR = 1.44; 95%CI = 1.13–1.84), and birth in a health facility (AOR = 1.22; 95%CI = 1.01–1.48) were all higher in women whose male partners’ involvement scores were higher.

Table 2 Logistic regression AOR for the association between male partners’ involvement and women’s utilization of maternal health care services, Addis Ababa, Ethiopia, 2014 (*N* = 210).

## Discussion

The study examined the role of male partners involvement in maternal health care as a determinant factor for female partners’ utilization of maternal health care services in a couple’s perspective.

Overall, male partners’ involvement in maternal health care services was quite low in the study. The proportion of male partners involved in at least seven of the eight activities was less than one in ten, which is comparable with similar studies on male partner’s involvement in PMTCT of HIV among male partners in Addis Ababa [43] and Arba Minch [46].

Although only a few had physically entered the ANC room, male partners accompanied their partner to ANC visits at least once in more than half of the study couples. This is higher than a report from Mekelle, where only 20% of pregnant women were accompanied by their male partners [45].

The study found out that proportions of male partners counseled and tested for HIV with their partners were about 20 %. This is lower than a recent study report in Addis Ababa, which showed that 60% of male partners tested for HIV [35], and a study in Gondar, reported that 40.1% of male partners received counseling and testing during their wife’s pregnancy [44].

The study has demonstrated a statistically significant association between male partners’ involvement in some activities and some of the maternal health care services use of their partners. Previous studies elsewhere also have linked male partners’ attendance to ANC with increased maternal health service utilization [29–31]. This could be explained by the fact that the active involvement of male partners makes them more aware of the significance of maternal health care services and support their partners [29]. Male partners’ awareness could also make them lenient in giving permission and provide necessary resources such as financial support for their partners’ maternal health care services.

Male partner’s involvement in women’s ANC visits has been shown in the literature that it may lead to increased male partners’ knowledge about women’s health care during pregnancy, therefore one could hypothesize that this knowledge could translate through pregnancy to childbirth, where utilization of maternal health care by their women may be increased [29–31].

Our findings are consistent with the findings of several studies in sub-Saharan Africa and elsewhere [18, 19, 29, 30, 34, 51], which suggested male partners’ involvement in maternal health care during pregnancy has benefits on maternal health care services access and utilization. The fact that the increase in male partner’s involvement increases their knowledge, and their attitude towards maternal health services becomes positive could be possible explanation for the association.

### Strength and limitation of the study

The study used a cross-sectional design, thus reverse causation may be a possible alternative explanation for associations and self-report might have also introduced social desirability bias. Despite this inherent limitation, the scales used in the study has high Cronbach’s alpha indicating an acceptable internal consistency. The study provides useful information on the impact of male partners’ involvement on maternal health care services utilization that will inform health service planners to design strategies to improve maternal health in Ethiopia.

## Conclusion

This study highlights a number of issues useful to understand the association between utilization of maternal health care services of female partners and their male partners’ involvement. Male partners’ involvement in maternal health care services showed significant associations with utilization of some of the maternal health care services by their partners. Future efforts to sustain and further improve the recent achievement in maternal health in the country should give due attention to male partners’ involvement and implement innovative strategies to reach out to all men with partners in the reproductive age.

## Abbreviations

ANC: antenatal care
AOR: adjusted odds ratio
EDHS: Ethiopian demographic and health survey
HEWs: health extension workers
HIV: human immunodeficiency virus
IPV: intimate partner violence
MDG: Millennium Development Goals
MTCT: mother-to-child transmission
PMTCT: prevention of mother-to-child transmission of HIV
SDG: Sustainable Development Goals
VCT: Voluntary Counseling and HIV testing
WHO: World Health Organization
